# Upper Airway Changes following Functional Treatment with the Headgear Herbst or Headgear Twin Block Appliance Assessed on Lateral Cephalograms and Magnetic Resonance Imaging

**DOI:** 10.1155/2019/1807257

**Published:** 2019-07-25

**Authors:** Min Gu, Fabio Savoldi, Urban Hägg, Colman P. J. McGrath, Ricky W. K. Wong, Yanqi Yang

**Affiliations:** ^1^Orthodontics, Faculty of Dentistry, The University of Hong Kong, Hong Kong; ^2^Department of Orthodontics, Dental School, University of Brescia, Brescia, Italy; ^3^Faculty of Dentistry, The University of Hong Kong, Hong Kong; ^4^Dental Public Health, Faculty of Dentistry, The University of Hong Kong, Hong Kong; ^5^Department of Dentistry and Maxillofacial Surgery, United Christian Hospital, Hong Kong

## Abstract

**Objective:**

The present study compared the changes in the upper airway dimensions and sleep-related breathing disorder (SRBD) condition between functional treatment with the headgear Herbst (HG-Herbst) and headgear Twin Block (HG-TB) appliance. Soft tissues were assessed on lateral cephalometric X-ray and magnetic resonance imaging (MRI).

**Materials and Methods:**

Consecutive patients who sought orthodontic treatment at the Faculty of Dentistry of The University of Hong Kong were screened. Adolescents (12-17 year sold for boys and 10-15 years old for girls), with class II molar relationship and overjet >5 mm, with no severe transverse maxillary deficiency, were recruited. Patients were assigned either to the HG-Herbst or to the HG-TB treatment by stratified block randomisation, with sex as the stratification factor. Lateral cephalograms, magnetic resonance imaging (MRI), and the Paediatric Sleep Questionnaire (PSQ) were obtained at baseline and after treatment.

**Results:**

28 patients were enrolled, and 26 patients (13 in each group) completed the treatment. Following 1 year of functional appliance treatment, a significantly lower increase of the lower anterior facial height was observed in the HG-Herbst group compared to the HG-TB group (*p* = 0.024). However, no significant differences were observed in the upper airway structures or SRBD between the two groups.

**Conclusion:**

The changes in upper airway dimensions and SRBD condition were not significantly different between the HG-Herbst and the HG-TB appliance treatment. Additional studies with larger sample size are warranted.

## 1. Introduction

A retrognathic mandible may be associated with narrowing of the upper airway and has been identified as a risk factor for childhood obstructive sleep apnoea (OSA) [1, 2]. Because functional appliances have long been used to treat children with mandibular retrognathism, they can potentially be beneficial for children with OSA as well [3, 4].

Functional appliances that position the mandible in a forward for the treatment of childhood OSA can be also considered as mandibular advancement devices (MADs) [3]. MADs are frequently used for the treatment of adult OSA since, by posturing the mandible forward, the device may enlarge the upper airway and improve the respiratory function. However, MADs in adults are effective only meanwhile they are* in situ* during sleep, whereas in children, MADs aim to produce a long-term improvement of OSA by stimulating mandibular growth [3]. Although insufficient evidence exists to support the use of functional appliances for treating childhood OSA [3, 4] studies focusing on their effects on the upper airway have shown encouraging results [5].

The Herbst and the Twin Block (TB) appliance are among the most commonly used functional appliances for stimulating mandibular growth. It is worth noting that different functional appliances may lead to variable effects on the dentoalveolar and skeletal structures [6], as well as on the upper airway [7]. For example, one study showed that the TB determined an increased posterior facial height compared to the Herbst [8].

Therefore, it is of interest to investigate whether this difference can result in different changes in the upper airway dimensions and sleep-related breathing disorder condition.

## 2. Materials and Methods

### 2.1. Subjects

**Figure 1:**
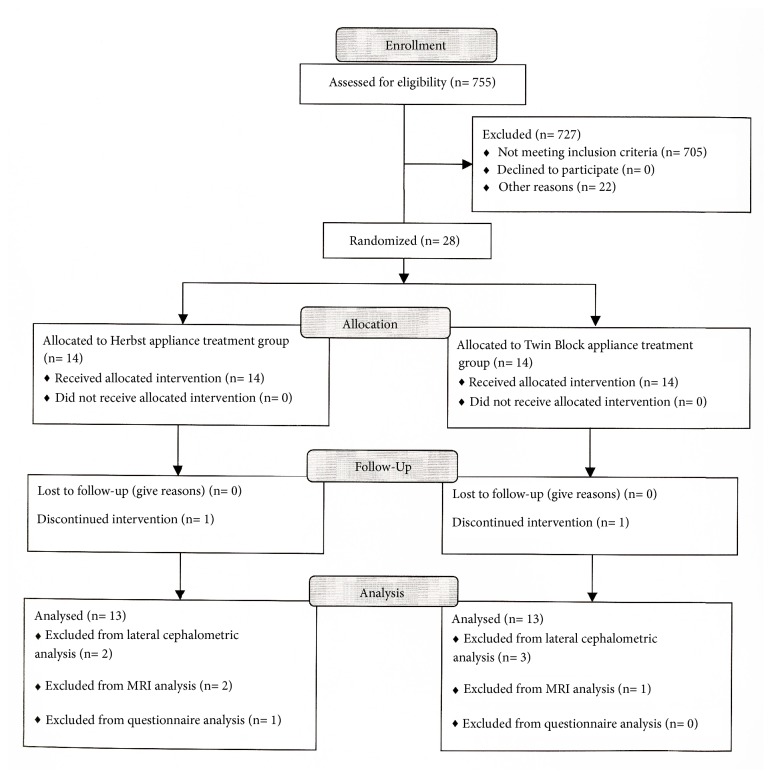
Trial flowchart.

755 consecutive patients who sought orthodontic treatment at the Faculty of Dentistry of The University of Hong Kong were screened. The inclusion criteria were 12-17 years old for boys and 10-15 years old for girls (ages at which the pubertal growth spurt occurs [9]), presenting bilateral class II molar relationship, and increased incisal overjet (> 5 mm). The exclusion criteria were cleft lip and palate, craniofacial syndromes, and severe transverse maxillary deficiency. Fifty adolescents fulfilled the inclusion criteria and 28 of them (11 boys and 17 girls) were assigned to functional appliance treatment followed by fixed appliance treatment (Figure 1 and Table 1).

After obtaining informed consent, the patients were randomly assigned to receive either the headgear Herbst (HG-Herbst) or the headgear Twin Block (HG-TB) appliance treatment. Stratified block randomisation (with block size of 4 and allocation ratio 1:1) was used, with sex as the stratification factor. One investigator (M.G.) conducted the allocation, and allocation concealment was achieved by anonymising the identity of the patients with a code.

The sample size could not be calculated a priori because of the lack of similar comparisons in the published literature.

The present study was approved by the Institutional Review Board of The University of Hong Kong/Hospital Authority Hong Kong West Cluster (IRB reference number: UW 12-405) and was registered at the US National Institutes of Health (ClinicalTrials.gov Identifier: NCT02448017). Informed consent was obtained from all the patients' parents in written format.

### 2.2. Treatment

Both the HG-Herbst and the HG-TB appliance used in the present study had an expansion screw for expanding the maxillary arch and two headgear tubes next to the maxillary premolars for attaching the high-pull headgear. Patients wore the high-pull headgear for 10 to 12 h/day, with a force of 500 g on each side. In some patients, palatal expansion was performed for the purpose of matching the upper and lower dental arches (device activation < 3 mm).

The initial mandible protrusive bites were taken preferably in an edge-to-edge position or, if not possible, in a maximum protrusive position that was comfortable for the patient. After six months of treatment, a second advancement was performed for the patients who had not yet reached the incisal edge-to-edge position. This reactivation was produced by adding acrylic to the HG-TB appliance, or by soldering a metal shim on the plunger of the HG-Herbst appliance. The treatment was planned to last for 1 year in both groups.

### 2.3. Lateral Cephalograms

Pre- and posttreatment lateral cephalograms were taken using the same X-ray machine (GE1000, General Electric, Milwaukee, WI, USA). The magnification was set to 12.5% for the mid-sagittal structures. The lateral cephalograms were obtained while patients maintained a natural head posture with the teeth in a light central occlusion. The patients were asked to breathe-in slowly and then exhale, holding the position and refraining from swallowing during exposure.

**Figure 2:**
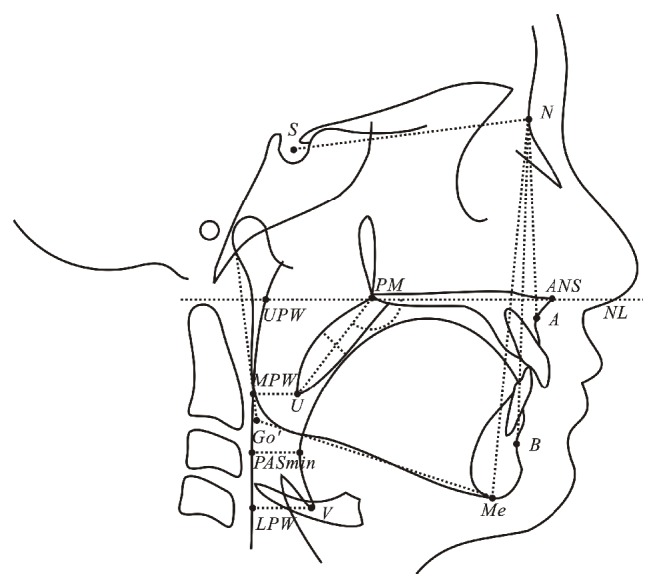
Cephalometric landmarks and measurements.

The upper airway was measured by using landmarks and reference lines (Table 2 and Figure 2). Cephalometric analysis was performed using cephalometric software (CASSOS, Soft Enable Technology Limited, Hong Kong SAR, PR China). The linear measurements were corrected according to the magnification.

Two patients in the HG-Herbst group showed swallowing actions during exposure, and three patients in the HG-TB group did not receive posttreatment lateral cephalograms. These data were not analysed (Figure 1).

### 2.4. Magnetic Resonance Imaging

MRI was performed at the Department of Diagnostic Radiology of The University of Hong Kong with a clinical 3.0T MRI system (Achieva 3.0T TX, Philips healthcare, Netherlands). The images of the head were acquired on the sagittal plane with a 3D T1 sequence (3D THRIVE sequence), 1 mm × 1 mm × 1 mm voxel size, 32 s scan time.

During scanning, awake patients were in the supine position and were asked to breathe normally through their nose, not to move their head, and to refrain from swallowing.

**Figure 3:**
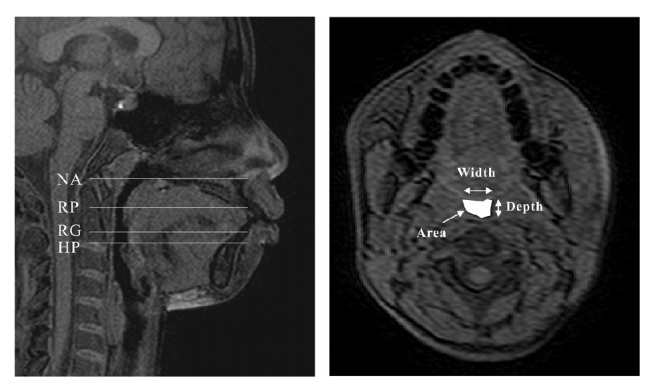
Landmarks and measurements of the upper airway on MRI. The nasopharynx (NA) was measured on the axial cross-section of the airway passing through the palatal plane. The retropalatal oropharynx (RP) was measured on the axial cross-section of the airway with the minimum area, between the palatal plane and the tip of the uvula. The retroglossal oropharynx (RG) was measured on the axial cross-section of the airway with the minimum area, between the tip of the uvula and the tip of epiglottis. The hypopharnx (HP) was measured on the axial cross-section of the airway passing through the tip of epiglottis.

The MRI images were measured using image-processing software (Mimics 14.1, Materialise, Leuven, Belgium). Before measurement, images were reoriented along the sagittal, axial, and coronal planes to standardise the head position. Measurements of the upper airway included depth, width and area at nasopharynx (NA), retropalatal oropharynx (RP), retroglossal oropharynx (RG), and hypopharnx (HP) (Figure 3).

### 2.5. Sleep-Related Breathing Disorder Scale

The Paediatric Sleep Questionnaire (PSQ) [10] is a parent-reported 22-item sleep-related breathing disorder (SRBD) questionnaire for screening OSA in children. Scoring is based on the percentage of “yes” answers, and a score of 33% is the cut-off value for OSA risk. The present study used the Chinese version of the PSQ [11], and, for patients whose score was > 33%, the family doctor was informed. However, during the study, none of the patients received treatment for OSA other than functional appliances.

### 2.6. Statistical Analysis

One orthodontist (MG) performed all measurements. Thirty randomly selected cephalograms and all MRI images were repeatedly measured at a two-week interval by the primary assessor (MG). The intraclass correlation coefficient (ICC) and the Dahlberg's formula [12] were used to assess the method error.

The Shapiro-Wilk test was used to evaluate the normality of the data distribution. According to the distribution, the two-sample *t*-test or the Mann-Whitney U-test was applied to assess inter-group differences. Intragroup differences between baseline and after treatment were assessed with the paired *t*-test or the Wilcoxon signed-rank test. The Pearson or the Spearman correlation coefficients were applied to determine the correlation between the changes in the upper airway and the PSQ score. The statistical significance was set at *p* < 0.05. Statistical analysis was performed using SPSS software (IBM SPSS Statistics 20, IBM Corp., US).

## 3. Results

### 3.1. Method Error

The ICC for single measurements ranged from 0.911 to 0.999 for lateral cephalometric analysis, and from 0.784 to 0.996 for MRI analysis.

The random errors for single measurements in lateral cephalometric analysis ranged from 0.4 to 0.8 mm for linear measurements and from 0.7° to 0.9° for angular measurements. In MRI analysis, it ranged from 0.5 mm to 2.1 mm for linear measurements and from 6.7 mm^2^ to 16.5 mm^2^ for area measurements.

### 3.2. Missing Data

During treatment, two patients (one in each group) dropped out from the study. The remaining 26 patients (9 boys and 17 girls) completed the treatment. During MRI three patients, two in the HG-Herbst group and one in the HG-TB group had obvious head movements and their data were not analysed. One posttreatment questionnaire was not returned in the HG-TB group (Figure 1). The characteristics of the included patients are presented in Table 1.

### 3.3. Intragroup Changes following Treatment

From lateral cephalometry, the RP depth (U-MPW, *p* = 0.005) increased significantly in the HG-Herbst group, and the soft palate thickness (SPT, *p* = 0.017) increased significantly in the HG-TB group compared to baseline, whereas the inclination of the soft palate (NL/PM-U, *p* < 0.001) decreased significantly in in both groups. Except for the SNA and the SN/MP angles, the other craniofacial parameters changed significantly in both groups after treatment. From MRI, no significant changes were present in the upper airway dimensions, and no differences were present in the PSQ scores in both groups, between pre- and posttreatment (Table 3). Moderate significant correlations were observed between the improvement of the PSQ and the increase in the U-MPW depth (*r *= 0.45,* p* = 0.047), the RP depth (*r* = 0.45,* p* = 0.040), and the HP area (*r* = 0.44,* p* = 0.043) (Table 4).

### 3.4. Comparison of Changes between HG-Herbst and HG-TB Groups

Following treatment, no significant differences were observed in the changes in the upper airway or PSQ scores between the two groups. However, the HG-Herbst group exhibited a lower increase in the lower anterior facial height (-1.6 mm, CI from -2.9 to -0.2,* p = *0.024) than the HG-TB group (Table 3).

## 4. Discussion

To investigate the changes in upper airway dimensions, 3D imaging is preferred to a 2D lateral cephalogram. The commonly used 3D techniques include cone beam computed tomography (CBCT) and MRI. CBCT provides similar accuracy and a lower dose of radiation than traditional spiral CT, but the radiation provided still limits its use in children [13]. MRI provides better soft tissue definition without exposing patients to ionising radiations but has the disadvantages of a higher cost and a longer examination time, which may result in motion artefacts [14].

In the present study, an MRI protocol with a quick scan time (32 s) was used, which was shorter than the 4 min scan time reported by previous studies [15, 16] but still longer than the 17 s reported for CBCT [17].

Although in the present study no significant difference was found between the two groups by comparing the MRI images, the moderate correlation between the improvement of PSQ score and the HP area suggests considering possible treatment effects on the soft component, which is better with MRI [16]. Future studies with a larger sample size may better clarify the role of MRI in assessing these changes in predictive terms as well.

In addition to the increase in the oropharyngeal depth, the increased thickness and the decreased inclination of the soft palate found in the present study were also favourable changes to the patency of the upper airway. When the mandible grows forward, the tongue is displaced anteriorly and moves away from the soft palate, which undergoes dimensional and angular changes [7]. These findings are consistent with those reported by previous studies [7, 18].

The present study also showed that the improvement of the PSQ score was associated with changes in the U-MPW depth, RP depth, and HP area, which may be the mechanism underlying the effects of the functional appliance during treatment of childhood OSA.

Although different functional appliances may produce different skeletal and dentoalveolar effects [6, 19], only a small difference was observed in their effects on the upper airway. Kinzinger et al. [20] compared the Herbst and the Functional Mandibular advance device, Godt* et al.* [21] compared the Harvold activator and the bite-jumping appliance, and the present study compared the Herbst and the TB appliances. None of these studies found significant differences between these appliances. Jena et al. [7] compared the TB appliance with the mandibular protraction appliance-IV (MPA-IV) and found a greater increase in the soft palate thickness in the TB group, which was also associated to an increase of the HP depth.

Among the appliances compared in the literature, the MPA-IV is the only one with inter-arch flexible force modules [22]. This appliance has been reported to produce fewer skeletal but more dentoalveolar effects compared with other functional appliances [19, 23]; consequently it may also produce fewer effects on the upper airway.

Although the primary objective of the present study was not to compare the craniofacial changes between the two treatments, the results were similar to those of Schaefer et al. [8]. In both studies, the TB appliance increased facial height more than the Herbst appliance. However, the present study showed that this effect did not result in differences in the upper airway.

Both the Herbst and the TB appliances have been reported to be effective in the treatment of childhood OSA [18, 24]. To the best our knowledge, this study was the first randomised controlled trial directly comparing them. The present study determined the effects of the Herbst and the TB appliances on the PSQ score and showed no significant difference in the improvement in the SRBD between the two appliances.

For ethical reasons, the present study did not include untreated controls, and the changes following treatment should be seen as the combination of growth and treatment. As a screening tool for childhood OSA, the findings from the PSQ should be further confirmed using polysomnography. Given also the limitations in the sample size, further research is required to confirm the present results.

## 5. Conclusions

The changes in upper airway dimensions and sleep-related breathing condition were not significantly different following the HG-Herbst and the HG-TB appliance treatments. Additional studies with larger sample size are warranted.

## Figures and Tables

**Table 1:** Demographic data of the study subjects.

	Total	HG-Herbst	HG-TB	
	(n)	(n)	(n)	
Boys	9	4	5	
Girls	17	9	8	
Total	26	13	13	

	Total	HG-Herbst	HG-TB	*p* value
	(mean ± SD)	(mean ± SD)	(mean ± SD)	

Age at baseline (year)	13.1 ± 1.5	13.5 ± 1.7	12.8 ± 1.3	0.128
Age after treatment (year)	14.2 ± 1.6	14.6 ± 1.9	13.8 ± 1.2	0.117
Duration of treatment (year)	1.1 ± 0.3	1.1 ± 0.2	1.1 ± 0.1	0.497

**Table 2:** Cephalometric landmarks and measurements of the upper airway and craniofacial structures.

Variable	Unit	Definition
*Landmarks*		
ANS		Anterior nasal spine, the tip of the median, sharp bony process of the maxilla
PM		Pterygo-maxillare, the point at the junction of the pterygo-maxillary fissure and the posterior nasal spine
U		Uvula, the tip of the uvula
UPW		Upper pharyngeal wall, the point of intersection of the line NL to the posterior pharyngeal wall
MPW		Middle pharyngeal wall, the point of intersection of the perpendicular line from U to the posterior pharyngeal wall
LPW		Lower pharyngeal wall, the point of intersection of the perpendicular line from V to the posterior pharyngeal wall
V		Vallecula, the point of intersection of the epiglottis and the base of the tongue
NL		Nasal line, the line joining ANS and PM
S		Center of the sella turcica
N		Nasion, the deepest point in the concavity of nasofrontal suture
A		A point, the deepest point in the concavity of the anterior maxilla between the anterior nasal spine and the alveolar crest
B		B point, the deepest point in the concavity of the anterior mandible between the alveolar crest and the pogonion
Me		Menton, the most inferior point on the body of the chin
Go'		Gonion' point, the point of intersection of the tangents of the inferior and posterior borders of the mandible
*Measurements*		
PM-UPW	mm	Depth of the nasopharyngeal airway space from PM to UPW
U-MPW	mm	Depth of the retropalatal oropharyngeal airway space from U to MPW
PASmin	mm	Depth of the retroglossal oropharyngel airway space, the shortest distance between the base of the tongue and the posterior pharyngeal wall
V-LPW	mm	Depth of the hypopharyngeal airway space from V to LPW
PM-U	mm	Length of soft palate, the distance from PM to U
SPT	mm	Soft palate thickness, the maximal thickness of the soft palate measured perpendicular to PM-U line
NL/PM-U	°	Inclination of the long axis of the soft palate relative to the nasal line
SNA	°	Angle between the S-N line and the N-A line
SNB	°	Angle between the S-N line and the N-B line
ANB	°	Angle between the N-A line and the N-B line
TAFH	mm	Total anterior facial height, the distance from N to Me
LAFH	mm	Lower anterior facial height, the distance from the intersection of N-Me line and NL line to Me
TPFH	mm	Total posterior facial height, the distance from S to Go'
MP/SN	°	Angle between the S-N line and Me-Go' line

**Table 3:** Changes in the upper airway (from latera cephalometry and MRI) and PSQ score following functional appliance treatment.

		Pre-treatment					Post-treatment					Δ			Δ			HG-Herbst vs. HG-TB	95% CI			*p* value
	n	HG-Herbst		HG-TB		*p* value	HG-Herbst		HG-TB		*p* value	HG-Herbst		*p* value	HG-TB		*p* value					
		Mean	SD	Mean	SD		Mean	SD	Mean	SD		Mean	SD		Mean	SD						
*Lateral cephalometry*																						
PM-UPW (mm)	21	24	3.3	22.8	3.7	0.449	25.0	2.5	23.0	4.0	0.171	1.1	1.7	0.068	0.2	2.1	0.796	0.9	-0.9	to	2.6	0.308
U-MPW (mm)	21	8.5	2.2	8.9	2.9	0.702	10.4	2.6	9.6	3.6	0.564	1.9	1.8	0.005*∗∗*	0.7	2.1	0.334	1.2	-0.5	to	3.0	0.161
PASmin (mm)	21	9.2	3.7	8.0	3.9	0.498	10.2	4.4	8.4	4.0	0.349	1.0	3.5	0.369	0.4	3.2	0.718	0.6	-2.4	to	3.7	0.679
V-LPW (mm)	21	16.3	2.8	14.7	2.5	0.168	17.5	2.9	14.2	1.7	0.005*∗∗*	1.2	1.8	0.055	-0.4	2.3	0.571	1.6	-0.3	to	3.5	0.093
PM-U (mm)	21	32.3	1.8	29.8	2.5	0.015*∗*	31.9	2.7	30.4	2.4	0.221	-0.5	2.7	0.570	0.6	2.6	0.477	-1.1	-3.5	to	1.3	0.360
SPT (mm)	21	7.2	1.0	6.9	1.2	0.541	7.5	1.5	7.7	1.5	0.810	0.3	1.1	0.389	0.8	0.8	0.017*∗*	-0.5	-1.4	to	0.4	0.298
NL/PM-U (°)	21	134.3	7.9	133.6	6.6	0.825	130.8	8.3	128.7	5.6	0.492	-3.5	3.3	0.006*∗∗*	-5.0	4.5	0.007*∗∗*	1.5	-2.1	to	5.1	0.404
SNA (°)	21	82.2	3.7	82.7	3.6	0.735	82.2	3.7	82.9	3.6	0.673	0.0	2.7	0.957	0.2	1.6	0.731	-0.2	-2.2	to	1.9	0.893
SNB (°)	21	76.2	2.7	77.6	3.5	0.321	78.5	2.4	80.2	3.5	0.214	2.3	1.5	<0.001*∗∗∗*	2.6	0.9	<0.001*∗∗∗*	-0.3	-1.4	to	0.9	0.630
ANB (°)	21	6	1.7	5.1	1.3	0.225	3.7	1.5	2.7	1.5	0.156	-2.3	1.6	0.001*∗∗*	-2.4	1.7	0.002*∗∗*	0.1	-1.4	to	1.7	0.848
TAFH (mm)	21	111.7	4.5	107.8	6.2	0.112	115.2	3.9	113.5	6.3	0.439	3.5	2.7	0.002*∗∗*	5.6	2.4	<0.001*∗∗∗*	-2.2	-4.5	to	0.2	0.071
LAFH (mm)	21	60.6	2.9	57.8	4.2	0.091	63.8	3.1	62.6	4.0	0.464	3.2	1.5	<0.001*∗∗∗*	4.8	1.5	<0.001*∗∗∗*	-1.6	-2.9	to	-0.2	0.024*∗*
TPFH (mm)	21	75.6	7.1	73.7	8.9	0.585	79.1	7.4	78.5	9.2	0.872	3.4	1.4	<0.001*∗∗∗*	4.8	1.9	<0.001*∗∗∗*	-1.4	-2.9	to	0.1	0.075
MP/SN (°)	21	34.2	6.7	32.2	5.5	0.465	33.7	8.2	32.0	5.8	0.581	-0.5	2.1	0.443	-0.2	1.5	0.720	-0.3	-1.9	to	1.3	0.690
*MRI*																						
NA width (mm)	23	24.6	4.3	23.8	3.9	0.413	26.0	3.8	24.3	4.1	0.260	1.4	4.9	0.477	0.5	2.3	0.328	0.9	-2.4	to	4.2	0.695
NA depth (mm)	23	12.7	4.0	12	3.5	0.740	14.2	4.1	12.9	3.2	0.449	1.4	2.8	0.182	0.9	1.6	0.182	0.6	-1.4	to	2.6	0.740
NA area (mm^2^)	23	251.7	115.7	225.9	66.4	0.976	289.9	111.3	246.2	76.0	0.347	38.3	84.1	0.213	20.3	43.5	0.158	18	-39.3	to	75.3	0.786
RP width (mm)	23	17.4	4.4	17.2	3.2	0.695	17.7	5.0	18.6	4.1	0.786	0.2	4.4	0.646	1.4	2.7	0.209	-1.2	-4.3	to	2.0	0.651
RP depth (mm)	23	7.7	2.8	7.2	2.1	0.608	8.0	2.6	8.0	2.3	0.928	0.3	1.4	0.534	0.9	1.8	0.071	-0.5	-2.0	to	0.9	0.413
RP area (mm^2^)	23	101.7	58.4	92.7	31.7	0.833	116.2	71.5	114.1	48.7	0.740	14.5	29.1	0.248	21.4	37.5	0.117	-6.9	-36.2	to	22.4	0.695
RG width (mm)	23	13.2	8.9	15.8	6.7	0.260	14.2	9.2	17.3	7.1	0.288	1.0	2.6	0.182	1.5	2.9	0.117	-0.5	-2.9	to	1.9	0.695
RG depth (mm)	23	12.3	3.6	12	3.6	0.608	12.1	3.5	11.8	3.7	0.413	-0.2	3.0	0.594	-0.1	1.8	0.906	-0.1	-2.2	to	2.0	0.608
RG area (mm^2^)	23	146.3	68.4	165.7	52.4	0.288	161.6	92.5	181.2	67.2	0.347	15.3	39.8	0.328	15.5	40.1	0.272	-0.1	-34.8	to	34.6	1.000
HP width (mm)	23	26.1	3.6	28.3	2.9	0.169	26.1	3.0	28.3	4.2	0.134	0.0	2.7	0.929	0.1	2.7	0.875	-0.1	-2.4	to	2.2	1.000
HP depth (mm)	23	12.6	3.8	12.2	2.9	0.880	12.6	4.0	12.3	2.3	0.786	0.0	2.1	0.594	0.1	1.5	0.929	-0.1	-1.7	to	1.5	0.651
HP area (mm^2^)	23	272.6	87.0	256.4	65.3	0.786	275.7	97.6	260.0	77.4	0.833	3.1	48.7	0.722	3.6	55.0	0.937	-0.5	-45.7	to	44.8	0.880
*PSQ*																						
Proportion	25	0.23	0.13	0.24	0.15	0.840	0.18	0.15	0.19	0.17	0.859	-0.05	0.09	0.078	-0.05	0.09	0.104	0.00	-1.4	to	1.8	1.00

*∗* = p < 0.05; *∗∗*= p < 0.01; *∗∗∗*= p < 0.001.

**Table 4:** Correlation analysis between changes in upper airway dimensions and PSQ score.

		Δ U-MPW depth	Δ RP depth	Δ HP area
		(mm)	(mm)	(mm^2^)
PSQ score	Coefficient	0.45	0.45	0.44
	*p* value	0.047*∗*	0.040*∗*	0.043*∗*

*∗* = p < 0.05

## Data Availability

The data used to support the findings of this study are available from the corresponding author upon request.

## References

[1] Flores-Mir C., Korayem M., Heo G., Witmans M., Major M. P., Major M. P. (2013). Craniofacial morphological characteristics in children with obstructive sleep apnea syndrome: a systematic review and meta-analysis. *Journal of the American Dental Association*.

[2] Katyal V., Pamula Y., Martin A. J., Daynes C. N., Kennedy J. D., Sampson W. J. (2013). Craniofacial and upper airway morphology in pediatric sleep-disordered breathing: systematic review and meta-analysis. *American Journal of Orthodontics and Dentofacial Orthopedics*.

[3] Nazarali N., Altalibi M., Nazarali S., Major M. P., Flores-Mir C., Major P. W. (2015). Mandibular advancement appliances for the treatment of paediatric obstructive sleep apnea: a systematic review. *European Journal of Orthodontics*.

[4] Carvalho F. R., Lentini-Oliveira D. A., Prado L. B., Prado G. F., Carvalho L. B. (2007). Oral appliances and functional orthopaedic appliances for obstructive sleep apnoea in children. *Cochrane Database of Systematic Reviews*.

[5] Xiang M., Hu B., Liu Y., Sun J., Song J. (2017). Changes in airway dimensions following functional appliances in growing patients with skeletal class II malocclusion: a systematic review and meta-analysis. *International Journal of Pediatric Otorhinolaryngology*.

[6] Phan K. L., Bendeus M., Hägg U., Hansen K., Rabie A. B. M. (2006). Comparison of the headgear activator and Herbst appliance—effects and post-treatment changes. *European Journal of Orthodontics*.

[7] Jena A. K., Singh S. P., Utreja A. K. (2013). Effectiveness of twin-block and Mandibular Protraction Appliance-IV in the improvement of pharyngeal airway passage dimensions in Class II malocclusion subjects with a retrognathic mandible. *The Angle Orthodontist*.

[8] Schaefer A. T., McNamara J. A., Franchi L., Baccetti T. (2004). A cephalometric comparison of treatment with the Twin-block and stainless steel crown Herbst appliances followed by fixed appliance therapy. *American Journal of Orthodontics and Dentofacial Orthopedics*.

[9] Hägg U., Taranger J. (1980). Skeletal stages of the hand and wrist as indicators of the pubertal growth spurt. *Acta Odontologica Scandinavica*.

[10] Chervin R. D., Hedger K., Dillon J. E., Pituch K. J. (2000). Pediatric sleep questionnaire (PSQ): validity and reliability of scales for sleep-disordered breathing, snoring, sleepiness, and behavioral problems. *Sleep Medicine*.

[11] Huang Y.-S., Wang C.-H., Guilleminault C. (2010). An epidemiologic study of sleep problems among adolescents in North Taiwan. *Sleep Medicine*.

[12] Dahlberg G. (1940). *Statistical Methods for Medical and Biological Students*.

[13] Al Najjar A., Colosi D., Dauer L. T. (2013). Comparison of adult and child radiation equivalent doses from 2 dental cone-beam computed tomography units. *American Journal of Orthodontics and Dentofacial Orthopedics*.

[14] Slaats M. A., Van Hoorenbeeck K., Van Eyck A. (2015). Upper airway imaging in pediatric obstructive sleep apnea syndrome. *Sleep Medicine Reviews*.

[15] Fregosi R. F., Quan S. F., Kaemingk K. L. (2003). Sleep-disordered breathing, pharyngeal size and soft tissue anatomy in children. *Journal of Applied Physiology*.

[16] Cappabianca S., Iaselli F., Negro A. (2013). Magnetic resonance imaging in the evaluation of anatomical risk factors for pediatric obstructive sleep apnoea–hypopnoea: a pilot study. *International Journal of Pediatric Otorhinolaryngology*.

[17] Iwasaki T., Takemoto Y., Inada E. (2014). Three-dimensional cone-beam computed tomography analysis of enlargement of the pharyngeal airway by the Herbst appliance. *American Journal of Orthodontics and Dentofacial Orthopedics*.

[18] Schütz T. C. B., Dominguez G. C., Hallinan M. P., Cunha T. C. A., Tufik S. (2011). Class II correction improves nocturnal breathing in adolescents. *The Angle Orthodontist*.

[19] Giuntini V., Vangelisti A., Masucci C., Defraia E., McNamara Jr J. A., Franchi L. (2015). Treatment effects produced by the Twin-block appliance vs the Forsus Fatigue Resistant Device in growing Class II patients. *The Angle Orthodontist*.

[20] Kinzinger G., Czapka K., Ludwig B., Glasl B., Gross U., Lisson J. (2011). Effects of fixed appliances in correcting Angle Class II on the depth of the posterior airway space: FMA vs. Herbst appliance—a retrospective cephalometric study. *Journal of Orofacial Orthopedics*.

[21] Godt A., Koos B., Hagen H., Göz G. (2011). Changes in upper airway width associated with Class II treatments (headgear vs activator) and different growth patterns. *The Angle Orthodontist*.

[22] McNamara Jr. J. A., Brudon W. L. (2001). *Orthodontics and Dentofacial Orthopedics*.

[23] Bilgiç F., Başaran G., Hamamci O. (2015). Comparison of Forsus FRD EZ and Andresen activator in the treatment of class II, division 1 malocclusions. *Clinical Oral Investigations*.

[24] Zhang C., He H., Ngan P. (2013). Effects of twin block appliance on obstructive sleep apnea in children: a preliminary study. *Sleep and Breathing*.

